# Characterization of Top Protein Candidates in Alzheimer's Disease in Down Syndrome, Early‐Onset, and Late‐Onset AD

**DOI:** 10.1002/alz70855_105910

**Published:** 2025-12-24

**Authors:** Mitchell Marta Ariza, Dominique Leitner, Evgeny Kanshin, Orrin Devinsky, Eleanor Drummond, Juan Fortea, Alberto Lleó, Beatrix Ueberheide, Thomas Wisniewski

**Affiliations:** ^1^ NYU Grossman School of Medicine, New York, NY, USA; ^2^ Institut de Neurociències, Universitat Autònoma de Barcelona, Bellaterra, Barcelona, Spain; ^3^ The University of Sydney, Sydney, NSW, Australia; ^4^ Sant Pau Memory Unit, Hospital de la Santa Creu i Sant Pau, Institut de Recerca Sant Pau ‐ Universitat Autònoma de Barcelona, Barcelona, Spain; ^5^ Center for Biomedical Investigation Network for Neurodegenerative Diseases (CIBERNED), Madrid, Madrid, Spain; ^6^ Catalan Foundation for Down Syndrome, Barcelona, Spain; ^7^ Sant Pau Memory Unit, Department of Neurology, Hospital de la Santa Creu i Sant Pau, Institut d'Investigació Biomèdica Sant Pau (IIB SANT PAU), Facultad de Medicina ‐ Universitat Autònoma de Barcelona, Barcelona, Spain; ^8^ CIBERNED, Network Center for Biomedical Research in Neurodegenerative Diseases, National Institute of Health Carlos III, Madrid, Spain; ^9^ New York University Grossman School of Medicine, New York, NY, USA

## Abstract

**Background:**

Down syndrome (DS) is the most common genetic form of Alzheimer's disease (AD) and is characterized by early amyloid‐β (Aβ) and phosphorylated tau (pTau) deposition, related to *APP* triplication. Our previous proteomics study comparing the Aβ plaque proteome in DS, early‐onset AD (EOAD), and late‐onset AD (LOAD) revealed multiple differentially abundant proteins linked to endo/lysosomal pathways, immune responses, and myelin function. While many of these proteins have been described in previous proteomics studies, several remain understudied in AD and DS, including some not previously reported in human AD proteomics. This study aims to characterize a selection of these proteins using immunohistochemistry to understand their roles in disease mechanisms, identify potential biomarkers, and explore novel therapeutic targets.

**Method:**

Immunohistochemistry (IHC) was performed on sections from the temporal and frontal cortex of DS, EOAD, LOAD, and age‐matched control cases. Proteins of interest were selected based on their abundance in Aβ plaques and surrounding non‐plaque tissue, limited characterization in AD and DS, and potential roles in AD neuropathology.

**Result:**

We selected the top altered proteins identified in Aβ plaques from our proteomics study for further characterization in DS, EOAD, and LOAD brain tissues. Our current work is the first to provide immunohistological evidence of these less‐studied proteins in the context of DS and AD. These proteins are associated with endo/lysosomal functions, synaptic activity, and myelin, and are linked to lysosomal storage diseases, protein aggregation, and the stability and maintenance of myelin sheaths.

**Conclusion:**

Our findings reveal key functional characteristics of the amyloid plaque proteome in DS, compared to EOAD and LOAD, and enhance our understanding of underexplored proteins that play pivotal roles in AD neuropathology beyond Aβ and Tau pathologies. These results could pave the way for follow up mechanistic studies, novel diagnostic biomarkers, and therapeutic strategies in both DS and AD by shedding light on previously understudied proteins.

